# Association Between Objectively Assessed Sleep and Depressive Symptoms During Pregnancy and Post-partum

**DOI:** 10.3389/fgwh.2021.807817

**Published:** 2022-01-31

**Authors:** Tryfonas Pitsillos, Anna-Karin Wikström, Alkistis Skalkidou, Birgit Derntl, Manfred Hallschmid, Nicolas D. Lutz, Edith Ngai, Inger Sundström Poromaa, Anna Wikman

**Affiliations:** ^1^Department of Women's and Children's Health, Uppsala University, Uppsala, Sweden; ^2^Department of Psychiatry and Psychotherapy, Tübingen Center for Mental Health, University of Tübingen, Tübingen, Germany; ^3^Lead Graduate School, University of Tübingen, Tübingen, Germany; ^4^Institute of Medical Psychology and Behavioral Neurobiology, University of Tübingen, Tübingen, Germany; ^5^German Center for Diabetes Research (DZD), Tübingen, Germany; ^6^Institute for Diabetes Research and Metabolic Diseases of the Helmholtz Center Munich at the University of Tübingen, Tübingen, Germany; ^7^Institute of Medical Psychology, Ludwig-Maximilians-Universität München, Munich, Germany; ^8^Department of Information Technology, Uppsala University, Uppsala, Sweden; ^9^Department of Electrical and Electronic Engineering, The University of Hong Kong, Hong Kong, Hong Kong SAR, China

**Keywords:** total sleep time, sleep efficiency, accelerometer, Edinburgh Postnatal Depression Scale, Sweden

## Abstract

**Introduction:**

Sleep problems are common in pregnancy but many studies have relied only on self-reported sleep measures. We studied the association between objectively measured sleep and peripartum depressive symptoms in pregnant women.

**Material and Methods:**

Sleep was assessed using Actiwatch accelerometers in a sample of 163 pregnant women in the late first (weeks 11–15) or early second trimester (weeks 16–19). Depressive symptoms were assessed in gestational weeks 17, 32 and at 6 weeks post-partum using the Edinburgh Postnatal Depression Scale (EPDS). Multiple linear regression and logistic regression analyses, adjusting for age, BMI, pre-pregnancy smoking, ongoing mental health problems, trimester and season of sleep assessment were carried out to test the association between sleep and depression. Sleep was measured by total sleep time and sleep efficiency, whereas depression was indicated by depressive symptoms and depression caseness. Results are presented as unstandardized beta (B) coefficients or adjusted odds ratios (AOR) and 95% confidence intervals (CI).

**Results:**

Total sleep time ranged from 3 to 9 h (mean 7.1, SD 0.9) and average sleep efficiency was 83% (SD 6.0). Women with the shortest total sleep time, i.e., in the lowest quartile (<6.66 h), reported higher depressive symptoms during pregnancy (week 17, *B* = 2.13, 95% CI 0.30–3.96; week 32, *B* = 1.70, 95% CI 0.03–3.37) but not post-partum. Their probability to screen positive for depression in gestational week 17 was increased more than 3-fold (AOR = 3.46, 95% CI 1.07–11.51) but unchanged with regards to gestational week 32 or 6 weeks post-partum. Sleep efficiency was not associated with depressive symptoms at any stage of pregnancy or post-partum.

**Discussion:**

In one of the few studies to use objective sleep measures to date, mental health of pregnant women appeared to be affected by shortened sleep, with total sleep time being negatively associated with depressive symptoms in the early second and third trimester. This finding highlights the relevance of identifying and treating sleep impairments in pregnant women early during antenatal care to reduce the risk of concomitant depression.

## Introduction

Pregnancy and the post-partum period are associated with an increased risk of developing mental health problems. The American Psychiatric Association's Diagnostic and Statistical Manual of Mental Disorders, fifth edition (DSM-5), specifies perinatal depression as a major depressive episode with onset during pregnancy or the first 4 weeks post-partum (1). However, as depression that begins later than 4 weeks after delivery or does not meet the full criteria for a major depressive episode may still cause harm and require treatment, experts in the field continue to define peripartum depression as a minor or major depressive episode occurring anytime during pregnancy or within the first year post-partum (2, 3). The condition affects ~10 to 15% of the global female population of childbearing age, with substantial variation across nations, partly explained by disparities in wealth inequality and maternal-child-health factors (4). Perinatal depression has major health and social impacts—not only for the becoming mother but also the offspring. Maternal suicidality and self-harm are crucial concerns among women who experience perinatal depression (5, 6). Furthermore, perinatal depression can lead to a variety of negative birth outcomes such as preterm birth, low birth weight, low APGAR score, and impaired breastfeeding (7–10). Perinatal depression also influences the mother's ability to parent and bond with the infant (11).

Multiple risk factors contributing to the development of perinatal depression have been identified, emphasizing the multidimensional nature of this condition. Socioeconomic factors such as maternal age, income, educational status, unemployment, smoking, domestic violence, an unsupportive partner, as well as a history of depression or other mental health conditions, hormonal factors and dysregulations of the HPA axis are possible determinants (10, 12–14). Across the majority of studies, a history of pre-pregnancy depression or mental health problems already at the start of pregnancy are typically the strongest risk factors for exacerbation of depression at any stage of pregnancy and the post-partum period (15, 16).

Pregnancy is commonly experienced to go along with increased sleep problems due to the tremendous anatomical, physiological and hormonal changes a pregnant woman's body undergoes (17–19). Typical self-reported pregnancy-related sleep problems include short sleep duration, poor sleep quality, sleep apnea, habitual snoring, and restless legs syndrome (20, 21). While many of these mostly subjective sleep problems are most prominent in the later stages of pregnancy—and may not necessarily be reflected by objective sleep measures relying on rigorous methodology [e.g., (22)]—many women complain of short sleep duration and poor sleep quality already in the first trimester (23–25). Sleep disturbances are also associated with depressive symptoms or clinical depression (26). In fact, impaired sleep is regarded as one of the most prominent symptoms in depressive patients. Notably, it is also a risk factor for developing depression, thus rendering sleep problems not just an epiphenomenon, but a predictor of depression (27). Common sleep disturbances related to depression are prolonged sleep latency, problems to maintain sleep, and early morning awakenings (26), thus implying a reduction of total sleep time. In fact, both short and long sleep duration has been suggested as an important factor in this context (28). Consequently, and in the context of pregnancy, sleep problems have been suggested to play a major role for the development of peripartum depression (29).

To date, there are few longitudinal studies demonstrating this relationship by using self-reported sleep problems during pregnancy as a predictor of later onset of depression. In one study four different sleep trajectories based on assessments at four time-points during pregnancy and up to 6 months post-partum were identified. The results indicated that women with the poorest sleep, with increasing severity of sleep problems during pregnancy, were more likely to develop depressive symptomatology in the post-partum period (30). Results from a large cohort study, collecting subjective sleep data during the second trimester of pregnancy, showed that poor sleep quality was positively correlated with more severe depressive symptoms during the second trimester and in the third month post-partum (31). One study evaluating the relationship between sleep quality in late pregnancy and recurrence of post-partum major depression through 28 weeks post-partum found poor sleep quality to predict recurrence after 4 weeks post-partum (32).

It should be pointed out that the majority of studies investigating sleep and perinatal depression have relied on subjective sleep assessments (using self-report questionnaires) and largely focused on sleep assessments in the third trimester and the post-partum period (19, 33, 34). However, the use of self-report may be problematic due to discrepancies between subjective and objective sleep measurements (22, 35), especially in patients with depression, who tend to over-report sleep problems (36, 37). Presently, only one study has used actigraphy for collection of objective sleep data in early pregnancy to study the influence of sleep deficiency on stress and depressive symptoms. Their results showed that sleep loss in the first and early second trimester was strongly associated with stress and depressive symptoms in early pregnancy (38).

Given the paucity of longitudinal studies investigating the interaction of sleep and depression in the peripartum phase, the aim of this study was to extend the knowledge on sleep problems in pregnancy and their association with concomitant and later development of depression. Specifically, we collected objectively measured (i.e., accelerometry-derived) sleep data between gestational weeks 11–19 and examined the association between total sleep time and sleep efficiency and perinatal depressive symptoms across three time-points during pregnancy and the post-partum.

## Materials and Methods

### Study Population

Data were obtained from (1) the Biology, Affect, Stress, Imaging and Cognition during pregnancy and post-partum (BASIC) study (39), and (2) the Safe Physical Activity in Pregnancy (SPAP) study, both carried out at the Department of Obstetrics and Gynecology, Uppsala University Hospital, Sweden.

Between September 2009 to November 2018 all pregnant, Swedish-speaking women of ≥18 years of age without confidential personal data, who were scheduled for a routine ultrasound (between weeks 16 and 19) in Uppsala County, received a letter with an invitation to participate in the BASIC study at their first ultrasound appointment. Women who agreed to participate returned their written informed consent by post. Participants were asked to complete online surveys at gestational weeks 17 and 32, and at 6 weeks post-partum. Since April 22nd 2016, women attending either the first trimester ultrasound examination between gestational weeks 11–15 or the second trimester ultrasound examination between gestational weeks 16–19 have also been invited to participate in the SPAP study. Participation involves wearing an accelerometer for physical activity and sleep recordings without interruption for seven consecutive days. Inclusion criteria were: an uncomplicated pregnancy at recruitment, ability to use a wrist watch during work hours and age 18 years or older. Written informed consent was obtained from all participants at inclusion. Upon inclusion, brief demographic data were collected including ongoing chronic health disorders, medication, smoking, and height and weight.

Between April 22nd 2016 and September 18th 2018, 174 women participated in both the SPAP and BASIC studies, of whom 11 had insufficient sleep data and were excluded. Thus, 163 women were eligible for inclusion in the present study. Ethical approval was granted by the Regional Ethical Review Board in Uppsala, Sweden ([1] Dnr 2009/171, and [2] Dnr 2016/142).

### Measures

#### Participant Characteristics

Information on participant characteristics was obtained from the standardized antenatal, obstetric and medical records and included age, parity (primiparous or multiparous), Body Mass Index (BMI) at first antenatal visit [categorized as <25 (underweight/normal weight) or ≥25 (overweight/obese) kg/m^2^], pre-pregnancy smoking (yes or no), trimester of sleep assessment (first or second), season of sleep assessment [November-February (winter) or March-October (not winter), based on seasonal differences in daylight hours]. Information on ongoing mental health problems was collected at the first antenatal visit and included self-reported ongoing depression, bipolar disorder, generalized anxiety disorder, panic disorder, attention deficit hyperactivity disorder, and personality disorder (categorized as presence of any ongoing mental disorder, yes or no).

#### Sleep Recordings and Sleep Parameters

Participants wore either an Actiwatch-2 or an Actiwatch Spectrum Plus accelerometer (Philips Respironics) on the non-dominant wrist without interruption for seven consecutive days. The Actiwatch-2 is a lightweight, water-proof solid state piezo-electric accelerometer with a silicon photodiode whereas the Actiwatch Spectrum Plus has a MEMS type accelerometer and a color sensitive photodiode which detects light with wavelength range 400–900 nanometers. In line with standard actigraphy provisions, the accelerometer detects movements of the wrist between 0.5 and 2.0 G, with a frequency response range between 0.35 and 7.5 Hz and a sampling frequency of 32 Hz. In comparison to polysomnography, which is the gold standard in sleep analysis, wearable sleep technology has been shown to be a reliable and validated method of assessing sleep characteristics (40, 41).

Participants were advised to stick to their everyday life during the study period. By pressing a button on the watch, participants recorded the time of going to bed in the evening and the time of waking up in the morning (to calculate time in bed). These events were used in the scoring algorithm to identify sleep periods, i.e., the interval between sleep onset and sleep offset. When the 7 days were completed, the accelerometer was returned by post and sleep data were extracted using Philips Actiware Software version 6.0.9. The amount of activity (number of movements in 30-s epochs and number of movements above a predefined threshold) was used for the determination of sleep and wake intervals. Individual actograms and sleep episodes were visually inspected before analyses to screen for adherence to the instructions, artifacts and malfunctioning. Participants were included in the analyses if they had a minimum of three valid sleep periods [the majority (87.7%) had valid recordings for seven nights]. The raw data from the actograms were then transferred to and analyzed in MATLAB (version 9.4), obtaining numeric values of the sleep parameters for statistical analyses.

Two of the most commonly studied sleep parameters were included for analyses in the present study; total sleep time and sleep efficiency. Total sleep time (hours) was calculated by excluding the sum of periods of wakefulness during the sleep period. Sleep efficiency is the ratio (in percent) of total sleep time and time in bed and reflects time spent awake in bed, including both sleep onset latency and wake after sleep onset.

#### Depressive Symptoms

Depressive symptoms at gestational weeks 17 (i.e., around the time of actigraphy recording) and 32 (i.e., in the third trimester), and at 6 weeks post-partum were assessed with the Swedish version of the Edinburgh Postnatal Depression Scale, EPDS (42, 43). The EPDS is a scale including 10 items each scored from 0 to 3 (total score between 0 and 30). Higher scores indicate more severe symptoms. Scores of ≥13 during pregnancy and ≥12 post-partum on the EPDS suggest clinically relevant depressive symptoms (i.e., screen-positive), as previously validated in Swedish perinatal samples (43, 44). The EPDS has shown adequate reliability and validity (45).

### Statistical Analyses

Descriptive statistics are reported as means and standard deviations (SD) or numbers (*n*) and percentages (%). Based on sample size and questions as to whether cut-offs from healthy populations are valid in pregnant samples, both sleep parameters were split in quartiles. To explore the relationship between total sleep time [defined as lowest quartile (<6.66 h) vs. the rest (≥6.66 h)] and sleep efficiency [defined as lowest quartile (<80% sleep efficiency) vs. the rest (≥ 80% sleep efficiency)] and depressive symptoms at gestational weeks 17 and 32, and at 6 weeks post-partum, three multiple linear regression analyses were carried out. The models adjusted for confounding by age, BMI at first antenatal visit, pre-pregnancy smoking, any ongoing mental health problems at first antenatal visit, trimester of sleep assessment, and season of sleep assessment. Parity was assessed as a potential confounder but did not influence either the exposure or the outcome and was thus not included in the models. Results are presented as unstandardized regression coefficients (B) with 95% confidence intervals (CI) including the *R*^2^ value for each analysis. In the final models, assumptions of multiple regression were met including no evidence of collinearity; all variance inflation factors were <2.0, and unstandardized residuals were normally distributed. In addition, we also performed multiple logistic regression analyses on risk of being screen-positive for depression (EPDS score ≥13 during pregnancy and ≥12 post-partum) using the same adjustments as in the multiple linear regression analyses. Statistical analyses were performed using IBM SPSS Statistics version 27.0 (IBM Corp., Armonk, N.Y., USA).

## Results

Participant characteristics are shown in Table 1. On average, women were 31.8 (SD 4.6) years old at inclusion and just over half of women were multiparous (*n* = 85, 52.1%). More than one-third was overweight or obese (*n* = 60, 36.8%) at first antenatal visit, and 7.4% reported pre-pregnancy smoking (*n* = 12). Approximately 14% reported ongoing mental health problems at the first antenatal visit (*n* = 23). In a majority of women, sleep assessment was carried out in the second trimester (*n* = 106, 65.0%), and in one-third of women during the winter months (*n* = 50, 30.7%).

**Table 1:** Participant characteristics (*n* = 163).

**Characteristic**	**Mean (SD) or *n* (%)**
Age, years	31.8 (4.6)
Parity
Primiparous	73 (44.8)
Multiparous	85 (52.1)
BMI (kg/m^2^), first antenatal visit	24.7 (4.7)
<25 (underweight/normal weight)	100 (61.3)
≥25 (overweight/obese)	60 (36.8)
Pre-pregnancy smoking
Yes	12 (7.4)
No	151 (92.6)
Ongoing mental health problems, first antenatal visit*
Yes	23 (14.1)
No	139 (85.3)
Trimester of sleep assessment
First	57 (35.0)
Second	106 (65.0)
Season of sleep assessment
Winter	50 (30.7)
Not winter	113 (69.3)

*BMI, Body Mass Index, at first antenatal visit; n, number of participants; SD, standard deviation*.

*Missing cases for parity n = 5 (3.1%), BMI n = 3 (1.9%), ongoing mental health problems n =1 (0.6%)*.

* *Including self-reported ongoing depression, bipolar disorder, generalized anxiety disorder, panic disorder, attention deficit hyperactivity disorder, and personality disorder*.

Total sleep time ranged from 3 to 9 h (mean 7.1, SD 0.9) and the average sleep efficiency was 83% (SD 6.0). Total sleep time and sleep efficiency were moderately and positively correlated (*r* = 0.48, *p* < 0.001). EPDS scores were available for 146 (89.6%) participants at gestational week 17, 148 (90.8%) women at gestational week 32, and 137 (84.0%) women at 6 weeks post-partum. Mean EPDS scores were 6.0 (SD 5.0), 5.7 (SD 4.7) and 5.6 (SD 4.6) at gestational week 17, 32 and at 6 weeks post-partum, respectively. The prevalence of clinically relevant depressive symptoms (i.e., screen-positive women) was 12.9% (*n* = 21) at gestational week 17, 8.6% (*n* = 14) at week 32, and 9.2% (*n* = 15) at 6 weeks post-partum.

Table 2 shows the results from the regression analyses. In gestational week 17, women with total sleep time in the lowest quartile reported significantly more pronounced depressive symptoms (adjusted for age, BMI at first antenatal visit, pre-pregnancy smoking, ongoing mental health problems, trimester of sleep assessment, and season of sleep assessment; *B* = 2.13, 95% CI 0.30–3.96) than women with total sleep time in the remaining quartiles. Sleep efficiency was unrelated to depressive symptoms at this time-point. In the adjusted model, older age was associated with fewer depressive symptoms (*B* = −0.22, 95% CI −0.40 to −0.04), whereas ongoing mental health problems at first antenatal visit were associated with more severe depressive symptoms (*B* = 3.74, 95% CI 1.52–5.97). In addition, women who completed sleep assessment during the winter months (November-February) reported more severe depressive symptoms (*B* = 2.01, 95% CI 0.23–3.80). The model explained 24% of the variance in depressive symptoms in gestational week 17. Table 3 shows the sleep variables in relation to the number of women who were screen-positive for depression at the different time-points. Women who had a total sleep time in the lowest quartile were more than three times more likely to be screen-positive for depression at this stage of pregnancy (OR = 3.46, 95% CI 1.07–11.51). Here, again, sleep efficiency was not associated with depressive symptoms.

**Table 2:** Multiple linear regression analyses of the association between objectively assessed sleep parameters and depressive symptoms on the EPDS at gestational weeks 17, 32 and 6 weeks post-partum adjusting for age, BMI at first antenatal visit, pre-pregnancy smoking, ongoing mental health problems at first antenatal visit, trimester at sleep assessment and season at sleep assessment.

	**Depressive symptoms gestational week 17**		**Depressive symptoms gestational week 32**		**Depressive symptoms post-partum week six**	
**Characteristics**	**Unstandardized regression coefficients, B (95% CI)**	***p***	**Unstandardized regression coefficients, B (95% CI)**	***p***	**Unstandardized regression coefficients, B (95% CI)**	***p***
Total Sleep Time (lowest quartile vs. the rest)	**2.13 (0.30–3.96)**	**0.03**	**1.70 (0.03–3.37)**	**0.05**	−0.63 (−2.37 to 1.12)	0.48
Sleep Efficiency (lowest quartile vs. the rest)	−0.48 (−2.47 to 1.52)	0.93	−1.40 (−3.28 to 0.49)	0.15	−1.09 (−3.09 to 0.91)	0.28
Age	–**0.22 (**–**0.40 to** –**0.04)**	**0.02**	−0.07 (−0.23 to 0.10)	0.43	−0.05 (−0.22 to 0.12)	0.54
BMI (overweight/obese vs. normal weight)	1.14 (−0.45 to 2.73)	0.16	**1.64 (0.21–3.06)**	**0.03**	0.78 (−0.72 to 2.29)	0.31
Pre-pregnancy smoking (yes vs. no)	2.55 (−0.51 to 5.60)	0.11	2.51 (−0.16 to 5.18)	0.07	**3.56 (0.86–6.25)**	**0.01**
Ongoing mental health problems (yes vs. no)	**3.74 (1.52–5.97)**	**0.001**	**5.50 (3.52–7.49)**	**<0.001**	**4.72 (2.75–6.68)**	**<0.001**
Trimester of sleep assessment (first vs. second)	0.58 (−1.24 to 2.40)	0.55	1.18 (−0.63 to 2.98)	0.20	0.32 (−1.62 to 2.27)	0.74
Season of sleep assessment (winter vs. not winter)	**2.01 (0.23–3.80)**	**0.03**	**1.81 (0.06–3.55)**	**0.05**	1.67 (−0.17 to 3.51)	0.08
*R* ^2^	0.24		0.31		0.25	

*CI, confidence interval; EPDS, Edinburgh Postnatal Depression Scale*.

*Statistically significant coefficients (p < 0.05) are marked in bold*.

**Table 3:** Multiple logistic regression analyses of the association between objectively assessed sleep parameters and being screen-positive for ongoing depression on the EPDS in gestational weeks 17, 32 and 6 weeks post-partum.

	**Screen-positive for depression gestational week 17**		**Screen-positive for depression gestational week 32**		**Screen-positive for depression post-partum week six**	
**Characteristics**	**AOR (95% CI)**	***p***	**AOR (95% CI)**	***p***	**AOR (95% CI)**	***p***
Total Sleep Time (lowest quartile vs. the rest)	**3.46 (1.07–11.51)**	**0.04**	1.18 (0.25–5.55)	0.84	0.66 (0.15–2.98)	0.59
Sleep Efficiency (lowest quartile vs. the rest)	0.75 (0.21–3.08)	0.76	0.32 (0.03–3.16)	0.33	0.25 (0.03–2.45)	0.23

*CI, confidence interval; EPDS, Edinburgh Postnatal Depression Scale*.

*Statistically significant adjusted odds ratios, AOR (p < 0.05), are marked in bold*.

*Models adjusting for age, BMI at first antenatal visit, pre-pregnancy smoking, ongoing mental health problems at first antenatal visit, trimester at sleep assessment and season of sleep assessment (winter vs. not winter)*.

In gestational week 32, adjusting for all other variables in the model, total sleep time in the lowest quartile (*B* = 1.70, 95% CI 0.03–3.37), overweight or obese BMI (*B* = 1.64, 95% CI 0.21–3.06), ongoing mental health problems at first antenatal visit (*B* = 5.51, 95% CI 3.52–7.49) and sleep assessment during the winter months (*B* = 1.81, 95% CI 0.06–3.55) were associated with depressive symptoms, whereas low sleep efficiency had no influence on depressive symptoms at week 32 (Table 2). The model explained 31% of variance in depressive symptoms in gestational week 32. However, none of the sleep variables influenced the risk of being screen-positive for depression in gestational week 32 (Table 3).

None of the sleep variables were associated with depressive symptoms at 6 weeks post-partum (Tables 2, 3). Instead, depressive symptoms in post-partum week six were predicted by ongoing mental health problems at the first antenatal visit (*B* = 4.72, 95% CI 2.75–6.68) and pre-pregnancy smoking (*B* = 3.56, 95% CI 0.86–6.25), after adjustment for all other variables in the model. The model in post-partum week six explained 25% of variance in depressive symptoms.

## Discussion

The main finding of this study was that pregnant women with sleep duration in the lowest quartile reported higher levels of depressive symptoms during pregnancy. This relationship was not only manifested at the time of the sleep recording, i.e., where it is unclear if the decreased sleep duration may be a symptom of ongoing depression or vice versa, but also later, in the third trimester of pregnancy, suggesting that short sleep durations may be a risk factor for developing depression. These findings were also independent of the women's age, BMI at first antenatal visit, smoking history, and the ongoing mental health problems some women reported at the first antenatal visit. Further, short sleep duration in the first or early second trimester of pregnancy was associated with increased risk of being screen-positive for depression in gestational week 17, but not at any later stage in pregnancy or post-partum. Finally, short sleep duration or poor sleep efficiency were not associated with depressive symptoms after childbirth.

Based on objective measurements, the findings of this study confirm prior findings of women's subjective reporting of sleep disturbances and their risk of developing depressive symptoms during pregnancy (30, 31, 33). Thus, this study has added important value to the subjective reports of women by establishing that poor sleep, in this case objectively evaluated by actigraphy, may contribute to an increased risk of depression during pregnancy. Women who had a total sleep time of <6.66 h before gestational week 20 reported on average 2.1 scale steps higher on the EPDS and were more than three times more likely to be screen-positive for depression in gestational week 17. This finding suggests that pregnant women who complain of short sleep duration in antenatal care should be further assessed for potential concomitant depression. We also found that women with sleep time <6.66 h reported on average 1.7 scale steps higher on the EPDS in gestational week 32, suggesting that sleep problems may maintain and prolong existing depressive symptoms or lead to development of depressive symptoms during pregnancy. The latter finding is also in line with meta-analytical approaches to disentangle the directional relationship between sleep disturbances and depression, where evidence at hand suggest that sleep problems, especially those reflecting wakefulness, precede the onset of depression. In contrast, little support has been found for a predictive role of depressive symptoms in the development of sleep disturbances (46). There were no association between total sleep time and being screen-positive for depression in gestational week 32, which may be explained by a lack of power to detect this association.

Although it could have been anticipated that sleep efficiency, which incorporates both sleep onset latency and wake after sleep onset (both of which are greatly affected in insomnia), would be an independent predictor of depressive symptoms in pregnancy, we did not find any relationship between objectively assessed sleep efficiency and depressive symptoms. Despite total sleep time and sleep efficiency being moderately and positively correlated shorter sleep durations for several consecutive nights may not necessarily imply poorer sleep efficiency, on the contrary, it might even increase sleep efficiency due to being constantly overtired. Multiple awakenings are common in the beginning of pregnancy, due to normal pregnancy-related causes such as nycturia, nausea, vomiting or pain, and hence, the association with depressive symptoms may be weakened (23–25). This study did not have the power to characterize sleep disturbances in pregnant women with depression, but future studies should aim to objectively describe the sleep problems that depressed women may suffer from to enhance our understanding of how to best help affected women.

Results of the present study were not in line with findings suggesting that poor sleep in pregnancy is associated with post-partum depression (33). This may be explained by the fact that studies on post-partum depression have carried out their sleep assessments in late pregnancy or post-partum, whereas women participating in the present study recorded their sleep patterns during the first half of pregnancy. These findings also emphasize that post-partum depression, in contrast to antenatal depression, likely comes with its own set of risk factors, where partner support, delivery complications, infant temperament and hormonal factors might play a more pivotal role (10). Further, sleep disturbances in the first half of pregnancy can have different causes than sleep disturbances in the post-partum period, meaning that some women who have unaffected sleep during pregnancy may very well-develop sleep disturbances after they have given birth.

While this study has added some information on the relationship between short sleep duration and depressive symptoms in pregnancy, it does not come without limitations. The major drawback of the study was the relatively low number of women who were screen-positive for depression. As recruitment targeted women scheduled for routine ultrasound assessment in pregnancy, the majority were healthy and at low risk of depression. This is reflected in the overall low mean EPDS scores at each time point, and in the relatively low proportion of women with ongoing mental health problems at the first antenatal visit. For this reason, we have limited power to answer some highly relevant questions like the impact on clinically relevant depression, and sleep characteristics in women with depression. Of course, it must be noted also that perinatal depression was assessed using self-report (i.e., the EPDS), and not by clinical interview. Further, as women had missing EPDS scores from some of the time-points, we refrained from evaluating how sleep impacts perinatal depression trajectories. Although many women were invited to participate we lack information on the response rate for the SPAP study. Thus, the study did not include a population-based sample, which may influence the generalizability of results. Further, it has recently been suggested that the interaction between insomnia, sleep apnea and circadian misalignment contribute to explaining the relationship between sleep deprivation and depression in pregnancy (47). However, as we lack information on some of these important aspects, we were unable to explore these factors as contributors to reduced total sleep in the present study. This, however, warrants further research in this population. Despite these limitations it is important to highlight that the major strength of the study is the objectively assessed sleep parameters.

## Conclusion

Based on objective measurements of sleep, the findings of this study confirm prior findings of women's subjective reporting of poor sleep and their risk of developing depressive symptoms during pregnancy. Although short sleep duration or poor sleep efficiency were not associated with depressive symptoms post-partum, many of the pregnant women included were affected by objectively measured poor sleep, in terms of shorter sleep duration associated with depressive symptoms in the early second and third trimester. These results point to the importance of addressing sleep impairments in pregnant women early during antenatal care which may attenuate the risk of concomitant depressive symptoms.

## Data Availability Statement

The raw data supporting the conclusions of this article will be made available by the authors, without undue reservation, upon reasonable request and with necessary ethics approval.

## Ethics Statement

The studies involving human participants were reviewed and approved by the Regional Ethical Review Board in Uppsala, Sweden (Dnr 2009/171, and Dnr 2016/142). The participants provided their written informed consent to participate in this study.

## Author Contributions

AS, IS, and A-KW: conceptualization and design. MH, NL, EN, TP, IS, and AW: data acquisition and analysis. AW and TP: drafting the work. All authors interpretation of data, revising the work critically for important intellectual content, and authors agreed to be accountable for all aspects of the work and approved the final version to be published.

## Funding

IS was supported by a grant from the Swedish Research Council 2020-01801. MH was supported by the German Federal Ministry of Education and Research (BMBF) to the German Center for Diabetes Research (DZD e.V.; 01GI0925).

## Conflict of Interest

Over the past five years, IS has served occasionally on advisory boards or acted as invited speaker at scientific meetings for Asarina Pharma, Bayer Health Care, Gedeon Richter, Peptonics, Shire/Takeda, and Sandoz. The remaining authors declare that the research was conducted in the absence of any commercial or financial relationships that could be construed as a potential conflict of interest.

## Publisher's Note

All claims expressed in this article are solely those of the authors and do not necessarily represent those of their affiliated organizations, or those of the publisher, the editors and the reviewers. Any product that may be evaluated in this article, or claim that may be made by its manufacturer, is not guaranteed or endorsed by the publisher.
